# Degradation-Aware Dynamic Kernel Generation Network for Hyperspectral Super-Resolution

**DOI:** 10.3390/s26041362

**Published:** 2026-02-20

**Authors:** Huadong Liu, Haifeng Liang, Qian Wang

**Affiliations:** School of Optoelectronic Engineering, Weiyang Campus, Xi’an Technological University, Xi’an 710021, China; 13998208070@163.com (H.L.); 2001310142@st.xatu.edu.cn (Q.W.)

**Keywords:** hyperspectral image, spectral super-resolution, dual-channel feature separation, spectral–spatial synergy, MSSCC Loss

## Abstract

Addressing the problems of the difficulty in reconstructing high-resolution hyperspectral images caused by dynamic degradation characteristics, the poor adaptability of traditional static degradation models, and the oversimplified noise modeling, this paper proposes a degradation-aware dynamic Fourier network (DADFN) for hyperspectral super-resolution. This method employs a dual-channel split module to decouple and encode spectral and spatial degradation information, realizes the independent mapping of spectral and spatial features via a multi-layer perceptron module, and integrates a spectral–spatial dynamic cross-attention fusion module to generate 3D dynamic blur kernels tailored to different bands and spatial positions. The proposed method designs a multi-scale spectral–spatial collaborative constraint (MSSCC) loss function to ensure the coordinated optimization of modeling rationality, spectral continuity, and spatial detail fidelity. Experiments on the CAVE and Harvard benchmark datasets demonstrate that the DADFN algorithm outperforms the baseline methods in all evaluation metrics, which proves the proposed method’s strong robustness in real-world complex degradation scenarios. This method provides a novel solution balancing physical interpretability and performance superiority for hyperspectral image super-resolution tasks and holds significant value for advancing its applications in remote sensing monitoring, precision agriculture, and other related fields.

## 1. Introduction

Hyperspectral images (HSIs) are rich in spatial and spectral information, thus holding great application value in remote sensing monitoring, environmental assessment, precision agriculture, and other related fields [1]. However, due to limitations in imaging equipment performance, atmospheric scattering interference, and transmission noise pollution, hyperspectral resolution (HR) images inevitably degrade, leading to low resolution (LR) [2]. Unlike RGB images, the degradation of HSIs exhibits significant dynamic characteristics in the spectral dimension—the degree and type of degradation vary distinctly across different bands, and the degradation parameters at different spatial positions within the same band also change dynamically with the spectral reflectance properties of the target scene [3]. It is precisely this dynamic degradation characteristic that makes spectral super-resolution (SSR) tasks extremely challenging.

In 1983, the U.S. Jet Propulsion Laboratory developed the first airborne imaging spectrometer, AIS-1 [4], which initially showcased the potential of hyperspectral remote sensing in geological and vegetation research. Since then, hyperspectral imaging technology has experienced significant progress. Although the origin of super-resolution technology can be traced back to the 1950s, it was not until the 1990s—with the advancement of digital image processing technology and the improvement of computer computing power—that it began to receive extensive attention and research [5]. The development of related algorithms has evolved from early traditional methods—including those based on operators and convolution sets [6,7,8,9] and those directly incorporating image degradation models [10,11,12,13]—to recent deep learning-based SSR algorithms [14,15,16,17]. Traditional SSR methods mostly rely on static degradation models, assuming that degradation parameters remain constant across the entire image—a simplification that makes them difficult to adapt to complex dynamic degradation scenarios. In recent years, deep learning has achieved breakthrough progress in the field of SSR. On one hand, by introducing a multi-scale feature fusion mechanism, it can better capture image information at different scales to improve super-resolution performance [18,19]; on the other hand, by enhancing the screening and fusion of cross-modal features and fully utilizing complementary modal information, it can effectively improve the quality of super-resolved images [20,21,22]. In addition, other studies have contributed to the development of this field from various dimensions. For example, the combination of the U-Net architecture and generative adversarial networks [16,23,24] has enhanced the model’s ability to restore details of HR images, and models based on spatial–spectral convolution [25,26] have made significant progress in SSR. Additionally, the introduction of attention mechanisms has strengthened the screening and fusion of cross-modal features, better utilizing the complementary information of different modal images to improve the quality of super-resolved images [27,28]. Nevertheless, existing deep learning models still have two shortcomings: first, the modeling method for degradation parameters such as blur kernels is fixed, making it impossible to dynamically adjust according to spectral characteristics; second, the assumption of noise distribution is overly simplistic, making it difficult to characterize the complex nature of mixed noise in HSIs [29].

To address the mentioned issues, this paper proposes a DADFN method. By dynamically modeling blur kernels, we developed a degradation-aware dynamic fusion network that adaptively estimates degradation model parameters in a data-driven manner, thereby providing a novel and effective solution for the practical application of HSI restoration technology.

The main contributions of this paper are as follows:

1. A dynamic blur kernel generation network module is designed. By splitting dual-channel latent variables, we achieve the decoupled encoding of spectral degradation information and spatial degradation information, enhancing the model’s ability for dynamic feature decoupling and fusion.
2. A dual-channel feature separation module is designed to decouple the spectral control sub-vector and the spatial control sub-vector. After decoupling, each spectral band corresponds to an independent feature channel, which is extended to the spatial band through spatial broadcasting + learnable weights; each spatial location also corresponds to an independent feature channel, which is extended to the spectral band through spectral broadcasting + learnable weights.
3. A spectral spatial dynamic cross attention integration module is designed to deeply combine dynamic kernel estimation with cross attention mechanisms, allowing spectral features to guide spatial kernel optimization. Utilizing spectral degradation information, it is possible to calculate attention weights, thereby adapting the kernel parameters of edge spatial positions to the spectral continuity of the corresponding band. At the same time, it enables spatial features to constrain spectral kernel adjustment, calculates attention weights using spectral degradation information, and adapts the kernel parameters of edge spatial positions to the spectral continuity of corresponding bands, achieving bidirectional enhancement of spectral information guided spatial features and spatial information guided spectral features.
4. A multi-scale spectral–spatial collaborative constraint (MSSCC) loss function was designed. Through the dynamically generated kernel total loss, multi-scale spectral total loss, and multi-scale spatial total loss, it ensures modeling rationality, spectral continuity, and spatial detail fidelity, and also enables the end-to-end optimization of degradation modeling and image restoration.

## 2. Related Work

### 2.1. Traditional SSR Algorithms

HSIs contain rich spatial and spectral information, enabling their wide application in astronomy, geography, meteorology, and military fields. Traditional HSIs are directly acquired by large-scale scanning of the earth’s surface using HSIs mounted on satellites or aircraft [30]. A notable limitation is that hyperspectral sensors are costly, which restricts their widespread adoption. Meanwhile, affected by sensor performance constraints and atmospheric interference, the acquired data are prone to noise contamination and spectral shifts. With the advancement of computer technology and the popularization of deep learning, it has become feasible to reconstruct high-spectral-resolution information from low-spectral-resolution data—driving SSR technology to emerge as a research hotspot. Traditional SSR methods can be categorized into two types: operator-based algorithms and degradation model-based algorithms.

#### 2.1.1. Operator-Based Methods

In 2017, Galliani et al. [31,32] pioneered spectral dimension super-resolution for HSIs, experimentally validating the feasibility and effectiveness of spectral domain convolution. Since then, with the advancing research frontier, an increasing number of heterogeneous operators have been tailored for SSR tasks, facilitating the continuous emergence of innovative research outcomes. As early as 2008, Parmar et al. [33] took the lead in leveraging sparse recovery to expand the spectral bands of RGB images, where HSIs were represented via sparse representation. Frosti Palsson et al. [34] proposed the integration of 3D convolutional operators into multispectral–hyperspectral image fusion, achieving SSR by jointly processing spectral and spatial dimensions through 3D convolutions. Liu D et al. [28] adopted group convolutional operators for spectral feature extraction, mitigating spectral distortion induced by conventional convolutions. Concurrently, they designed a spectral attention mechanism to adaptively recalibrate feature responses and incorporated spectral prior information to enhance the performance of SSR. Nevertheless, such operator-based SSR methods suffer from inherent limitations: first, the design of effective operators imposes high technical thresholds; second, the stacking of multiple operators tends to induce complexity accumulation, thereby increasing computational overhead; and third, these methods exhibit inadequate robustness to complex degradation scenarios.

#### 2.1.2. Degradation Model-Based Methods

In the field of SSR, traditional degradation models laid an important foundation for early research. While their limitations have gradually become prominent with technological advancements, they still offer valuable insights for subsequent studies. Among these paradigms, the bicubic degradation model stands as one of the earliest widely adopted frameworks. It obviates the need for estimating the complex degradation parameters, characterized by a straightforward model construction and solution workflow that enables efficient implementation. Nevertheless, due to its over-simplification of the underlying degradation mechanism, this model fails to accurately characterize practical imaging artifacts such as noise and atmospheric interference, neglects the inherent inter-spectral correlation, and is prone to inducing non-negligible spectral distortion during the reconstruction process [20].

To address the over-simplification limitation of the bicubic model, researchers proposed the linear blur–downsampling model. By incorporating a blur kernel into the mathematical formulation to simulate the point spread function of the optical system, this model aligns more closely with the physical imaging process. Furthermore, it explicitly accounts for noise components, thereby enhancing the model’s robustness [21]. However, the blur kernel in practical degradation scenarios exhibits inherent spatial variability and scene dependence, rendering a fixed kernel inadequate for accurately modeling real-world degradation. Moreover, the need to estimate both the blur kernel and noise parameters from LR images gives rise to an ill-posed inverse problem [35].

Leveraging the strong correlation and inherent redundancy of HSIs in both spatial and spectral dimensions, Xu, H. et al. [23] proposed a degradation model integrating low-rank and sparse priors. This model characterizes spectral correlation via low-rank constraints, preserves spatial edge structures through sparse regularization, and separates signal components from noise interference by means of low-rank decomposition—thereby enhancing the robustness of reconstruction. However, this method involves large-scale matrix decomposition and tensor computations, leading to substantial computational overhead. Furthermore, the intrinsic rank of practical HSIs is challenging to estimate accurately; additionally, solution approaches such as the truncated nuclear norm belong to non-convex optimization paradigms, which entail high computational complexity and thus fail to meet the requirements for real-time processing.

### 2.2. Deep Learning-Based SSR

With the development of deep learning in the field of computer vision, its application in SSR has become increasingly widespread. The enhanced deep super-resolution (EDSR) model, proposed by a research team from Seoul National University in South Korea in 2017 [24], optimizes the residual network structure to improve the reconstruction accuracy of LR images to HR images. Kuriakose, B. M. et al. [25] introduced a residual mechanism on the basis of the original EDSR model to improve robustness to outliers. Wu, G. and Jiang, J. [22] proposed the residual channel attention network (RCAN) model and integrated RCAN with transformer architecture. On the basis of deep residual networks such as EDSR, an attention mechanism is introduced to address the problem of different importance levels of features in different channels, achieving a significant improvement compared with EDSR for images with rich details. Li, J et al. [36] explicitly modeled the interdependencies between channels using an adaptive weighted attention network; Huang, Y. et al. [26] pre-trained the model using spectral response degradation loss and transferred it to a new spectral dataset. Y. Zhang et al. [37] creatively introduced spatiotemporal blocks, alternating between spatial and temporal attention, and integrated an adaptive condition injector with a spatiotemporal perception modulator. Deep learning-based methods possess powerful feature learning and non-linear mapping capabilities, leading to significant progress in SSR. However, they suffer from issues such as complex networks, lack of physical interpretability, and poor adaptability to complex degradation scenarios.

Subsequently, by combining physical models and embedding them into deep learning networks, variational models that jointly learn deep prior regularization and spectral degradation physical models have emerged. These methods make full use of data priors and explicitly solve the image degradation process. For example, Cai, Y. et al. [38] established a degradation model based on a transformer network, implicitly estimating information parameters from the degraded compressed measurements and the physical masks used in modulation. Yang, P. et al. [27] proposed the deep blind super-resolution (DBSR) model, which integrates the features of the original blurred image to correct any errors that may arise from the previous kernel estimation; Park, J. et al. [39] proposed an unsupervised blind super-resolution kernel estimation method (KernelGAN). By using a generative adversarial network to learn the degradation kernel of LR images, blind super-resolution reconstruction can be achieved without an external dataset. However, improvements are still needed in terms of noise robustness and dynamic scene processing.

Most of the current deep learning-based methods for SSR adopt static blur kernels, resulting in fixed degradation parameters during modeling and the inability to dynamically adjust according to spectral characteristics, which fails to truly reflect spectral characteristics in different scenarios. Furthermore, the assumption of noise is overly simplistic, making it difficult to describe the complex characteristics of mixed noise in HSIs.

To address the limitations of traditional degradation models, this paper proposes an improved framework that integrates an online blur kernel estimation module and a noise distribution prediction module. These dual modules dynamically adapt to real-world degradation scenarios, enabling accurate SSR.

## 3. Methods

In the degradation process of HSIs, spectral degradation and spatial degradation are inherently independent physical processes with weak inter-dimensional coupling. Traditional degradation models adopt fixed noise distributions and blur kernels, which fail to map latent variables across different dimensions to physical degradation attributes—such as spatial blur intensity and spectral coupling degree. This misalignment renders traditional models incapable of accurately characterizing the intrinsic degradation mechanism of HSIs. This paper designs a *split* (∙) [40] operator to explicitly disentangle spatial and spectral information embedded in latent variables, thereby decoupling the mixed spectral degradation cues and spatial degradation attributes within the low-dimensional latent space and enabling precise modulation of these two independent degradation processes. Furthermore, a cross-attention fusion module is proposed to establish inter-dimensional correlations via network learning, realizing adaptive attention recalibration along the feature dimension and enhancing the accurate characterization of the HSI degradation mechanism. Additionally, a hybrid loss function integrated with physical constraints is developed, which incorporates spatial detail preservation, spectral fidelity, and edge structure consistency. This loss function achieves dynamic weight adjustment to comprehensively optimize the trade-off between multi-dimensional performance metrics.

### 3.1. Degradation Model

The traditional spectral image degradation model assumes that all bands share the same degradation process [41], and its mathematical formula is expressed as:

(1) $$Y=\left(X⊗k\right){↓}_{s}+n$$

where X = HR image, **k** = Gaussian blur kernel, ${↓}_{s}$ = downsampling operation (hyperparameter s), and **n** = additive white Gaussian noise.

The degradation process is defined as follows: the HR image X is convolved with a Gaussian blur kernel **k**, the blurred image is then downsampled by a hyperparameter s, additive white Gaussian noise is subsequently added to the degraded image, and, finally, the LR image Y is obtained after compression.

The traditional degradation model uses fixed noise (represented as gray values) when dealing with blur degradation [11,42]. However, the degradation process in real scenarios is complex, where the blur kernel ***k*** and noise distribution parameters (λ, σ) are unknown and dynamically change. This leads to a decline in reconstruction performance when traditional degradation models with static blur kernels and fixed noise are used.

### 3.2. DADFN

Image degradation in real scenarios is not dominated by a single, fixed pattern but is dynamically influenced by multiple hyperparameters. To address the issue of reduced spectral reconstruction performance caused by fixed blur kernels in traditional degradation models, this paper proposes a DADFN model based on a dynamic blur kernel generation network in the spectral dimension.

By jointly modeling the coupling effect of spectral blur and spatial blur, let ***X*** be the original high-quality spectral image ($X\in R^{H\times W\times B}$) and Y be the low-quality image output after degradation. A spectral–spatial 3D dynamic convolution blur kernel $K\in R^{k\times k\times B\times B}$ is designed with an adaptive degradation parameter estimation module for each spectral band. This module dynamically predicts the blur kernel $k_{b}$ and noise parameter ***σ*** and embeds them into the degradation model to achieve end-to-end joint optimization:

(2) $$Y=D\left(X;\theta_{d}\right)+n\left(\theta_{n}\right)$$

where D (∙) is a dynamic degradation operator, representing the blur process controlled by the blur kernel parameter $\theta_{d}$, with the core being the application of spectral–spatial coupled blur to X; $n\left(\theta_{n}\right)$ denotes noise defined by the noise distribution parameter $\theta_{n}$, such as Gaussian noise, Poisson noise, etc.; $\theta_{d}$ is a parameterized representation of the 3D dynamic convolution blur kernel $K\in R^{k\times k\times B\times B}$; and $\theta_{n}$ is the noise distribution parameter.

**Figure 1:**
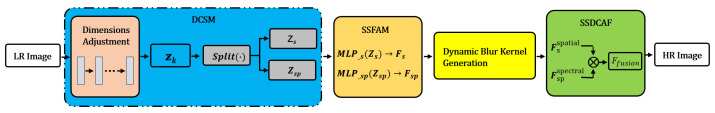
The architecture of the proposed DADFN.

It should be noted that in the process of generating dynamic noise, this article still uses the method proposed in reference [41], combined with the dynamic kernel fitted by the proposed network, and based on Equation (1), the SSR results of LR images can be obtained. The process of the method proposed in this article is shown in Figure 1.

#### 3.2.1. Dual-Channel Split Module (DCSM)

Assume that the blur kernel is controlled by a low-dimensional latent variable $z_{k}\in R^{m}$ and is generated by a neural network $f_{\phi }$:

(3) $$z=f_{\emptyset }\left(z_{k}\right),z_{k}\sim p(z_{k})$$

where $f_{\emptyset }$ represents the kernel generation network and $p\left(z_{k}\right)$ denotes the prior distribution of the latent variable, which adopts a uniform distribution or Gaussian distribution here.

**Figure 2:**
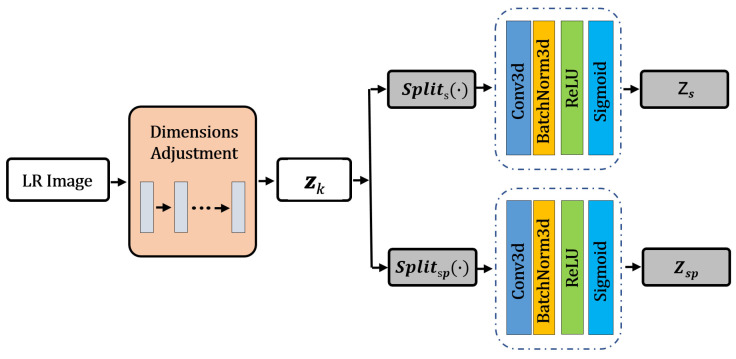
Dual-channel split module.

In the dynamic parameterization of the blur kernel, the core function of the neural network $f_{\emptyset }$ is mapping the low-dimensional latent variable $z_{k}$ to high-dimensional blur kernel parameters. The traditional approach directly upsamples the latent variable into fixed-size features, ignoring the differences in the control of spectral and spatial degradation by different dimensions of the latent variable. In response to this, this paper proposes an optimization method for traditional latent variables based on dual-channel feature separation. The flow of dual-channel feature separation is shown in Figure 2.

The LR HSI is used as the input. After dimension adaptive adjustment, the *split* (∙) operator splits the total latent variable $z_{k}$ into two independent sub-vectors $z_{s}$ and $z_{sp}$, each with a dimension of *m*/2, through a convolution network. These two sub-vectors have no shared parameters and undergo parallel and independent processing, ensuring the decoupled encoding of spectral degradation information and spatial degradation information. Let the total latent variable be $z_{k}\in R^{m}$ (where m is the total dimension). It is split into a spectral control sub-vector and a spatial control sub-vector, which are then output after 3D convolution.

The spectral control sub-vector is expressed as:

(4) $$z_{s}=Conv3d({split}_{s}\left(z_{k}\right))\in R^{m/2}$$

where *split* (∙) represents the extraction of the first *m*/2 dimensions from the total latent variable $z_{k}$ as the spectral control sub-vector.

The spatial control sub-vector is expressed as:

(5) $$z_{sp}=Conv3d({split}_{sp}\left(z_{k}\right))\in R^{m/2}$$

where *split* (∙) represents the extraction of the first *m*/2 dimensions from the total latent variable $z_{k}$ as the spatial control sub-vector.

#### 3.2.2. Spectral–Spatial Feature Alignment Module (SSFAM)

**Figure 3:**
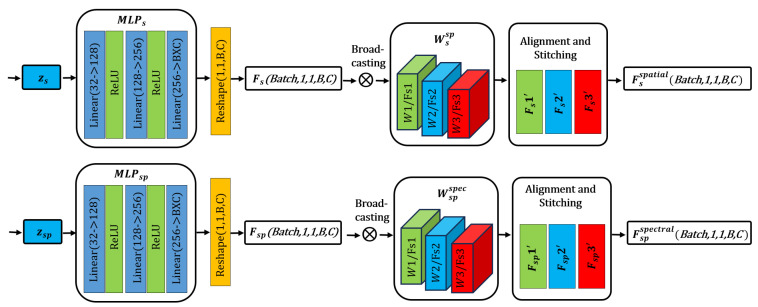
Spectral–spatial feature alignment module.

Two MLPs with no shared parameters are used to map the sub-vectors to spectral features and spatial features, respectively. The mapping process of the independent MLPs is shown in Figure 3.

In the spectral feature mapping ($F_{s}$), let the spectral MLPs be ${MLP}_{s}$, which consists of multiple fully connected layers and non-linear activation functions. The input is $z_{s}$, and it first outputs a high-dimensional vector, ${vec}_{s}={MLP}_{s}(z_{s})\in R^{1\times 1\times B\times C}$, which is then reshaped into a tensor of a specified dimension, $F_{s}=Reshape({vec}_{s})\in R^{1\times 1\times B\times C}$. Here, B is the number of spectral bands, C is the number of feature channels, and *Reshape* (∙) represents reshaping the vector into a 4D tensor of 1 × 1 × *B* × *C*.

In the spatial feature mapping ($F_{sp}$), similar to the spectral feature mapping, let the spatial MLPs be ${MLP}_{sp}$, which also consists of fully connected layers and activation functions. The input is $z_{sp}$, and it first outputs a high-dimensional vector, ${vec}_{sp}={MLP}_{sp}(z_{sp})\in R^{k\times k\times 1\times C}$, which is then reshaped into a tensor of a specified dimension, $F_{sp}=Reshape({vec}_{sp})\in R^{k\times k\times 1\times C}$. Here, k is the spatial kernel size, and the reshaped tensor has a dimension of k × k × 1 × C. From the above derivation, the latent variable feature enhancement formula can be obtained as follows:

(6) $$\left\{\begin{array}{c} z_{s},\;z_{sp}\leftarrow \mathrm{split}(z_{k})(z_{k}\in R^{m};\;z_{s},z_{sp}\in R^{m/2})\\ F_{s}\leftarrow {MLP}_{s}\left(z_{s}\right)\left(F_{s}\in R^{1\times 1\times B\times C}\right)\\ F_{sp}\leftarrow {MLP}_{sp}\left(z_{sp}\right)\left(F_{sp}\in R^{k\times k\times 1\times C}\right) \end{array}\right.$$

where the first two dimensions are spatial dimensions, the third is the spectral dimension, and the fourth is the feature channel dimension.

In order to spatially extend the spectral feature map $F_{s}$ with a spatial dimension of 1 × 1 to match the k × k spatial dimension of the spatial–spectral feature map $F_{sp}$, a spatial broadcast + spectral perception weight mechanism is adopted. The implementation method is as follows:

(7) $$F_{s}^{spatial}=F_{s}⨂W_{s}^{sp}\in R^{k\times k\times B\times C}$$

where ⨂ represents the broadcasting operation, which copies the 1 × 1 spatial dimension to a k × k spatial dimension. $W_{s}^{sp}\in R^{k\times k\times B\times C}$ is a learnable spectral–spatial weight. Dynamic feature expansion is achieved through the learnable spectral–spatial weight.

Then, a spectral broadcasting + spatial-aware weight mechanism is used to assign a spectral dimension to $F_{sp}$, realizing the spectral expansion of spatial features to match the B bands in the spectral dimension. The implementation method is as follows:

(8) $$F_{sp}^{spectral}=F_{sp}⨂W_{sp}^{spec}\in R^{1\times 1\times B\times C}$$

where $W_{sp}^{spec}\in R^{1\times 1\times B\times C}$ is a learnable spatial–spectral weight. Through the above steps, the spectral degradation control information and spatial degradation control information mixed in the low-dimensional latent variable are decoupled and aligned, resulting in clear single-dimensional features. Finally, by training the learnable.

#### 3.2.3. Spectral–Spatial Dynamic Cross-Attention Fusion Module (SSDCAF)

**Figure 4:**
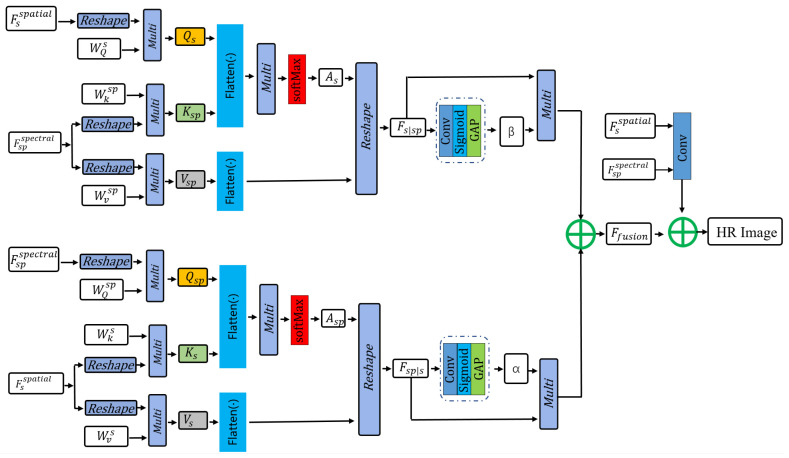
Spectral–spatial dynamic cross-attention fusion module.

Traditional spectral information encoding methods have the limitations of ambiguous physical interpretability, difficulty in decoupling coupled modeling, and encoding redundancy. To address these issues, this paper adopts a dual-channel feature separation approach to achieve dedicated encoding of spectral degradation information and spatial degradation information. Based on the aforementioned latent variable splitting independent mapping feature dimension adaptation framework, a spectral–spatial dynamic cross-attention fusion module is designed. The core of the proposed cross-attention fusion lies in establishing correlations between spectral features $F_{s}$ and spatial–spectral features $F_{sp}$ and calculates the cross-attention weights to realize bidirectional enhancement, where spectral information guides spatial feature refinement and spatial information guides spectral feature enhancement. Finally, through end-to-end training, the fusion of spectral and spatial features is adaptively learned, enabling the restoration of super-resolved HSIs. The implementation steps are illustrated in Figure 4.

Step1: Calculate the influence intensity of each band on each spatial position to realize the weight assignment of spatial features guided by spectral features:

(9) $$\left\{\begin{array}{c} Q_{s}=F_{s}^{spatial}\cdot W_{Q}^{s}\in R^{k\times k\times B\times C^{\prime }}\;\\ K_{sp}=F_{sp}^{spectral}\cdot W_{K}^{sp}\in R^{k\times k\times B\times C^{\prime }}\\ V_{sp}=F_{sp}^{spectral}\cdot W_{V}^{sp}\in R^{k\times k\times B\times C^{\prime }} \end{array}\right.$$

where $W_{Q}^{s}$, $W_{K}^{sp}$, and $W_{V}^{sp}$ are learnable weights.

Flatten the spatial and spectral dimensions into a sequence k×k×B=N, and calculate the global attention weight:

(10) $$A_{s}=softmax(\frac{Flatten(Q_{s})\cdot Flatten{\left(K_{sp}\right)}^{T}}{\sqrt{C^{\prime }}})\in R^{N\times N}$$

where $A_{s}[n_{1},n_{2}]$ represents the influence weight of the n1-th spatial + spectral feature position on the n2-th position.

Finally, weight the value vector $V_{sp}$ using the weight $A_{s}$ and reshape it to the original dimension:

(11) $$F_{s|sp}=Reshape(A_{s}\cdot Flatten(V_{sP}))R^{k\times k\times B\times C^{\prime }}$$

Step 2: Similar to Step 1, calculate the constraint intensity of each spatial position on each band to realize the weight assignment of spectral features constrained by spatial features:

(12) $$\left\{\begin{array}{c} Q_{sp}=F_{Ps}^{spectral}\cdot W_{Q}^{sP}\in R^{k\times k\times B\times C^{\prime }}\\ K_{s}=F_{s}^{spatial}\cdot W_{K}^{s}\in R^{k\times k\times B\times C^{\prime }}\\ V_{s}=F_{s}^{spatial}\cdot W_{V}^{s}\in R^{k\times k\times B\times C^{\prime }} \end{array}\right.$$

where $W_{Q}^{sP}$, $W_{K}^{s}$, and $W_{V}^{s}$ are the learnable weights.

Flatten and calculate the weight:

(13) $$A_{sP}=softmax(\frac{Flatten(Q_{sP})\cdot Flatten{\left(K_{s}\right)}^{T}}{\sqrt{C^{\prime }}})\in R^{N\times N}$$

Update the spectral features constrained by space:

(14) $$F_{sp|s}=Reshape(A_{sP}\cdot Flatten(V_{s})){\in R}^{k\times k\times B\times C^{\prime }}$$

Step 3: Design a dynamic gating mechanism for adaptive fusion and add residual connections to retain the original feature information, and then output the spectral–spatial joint feature. The process is as follows:

Let α and β be the weights controlling the spectral-dominated features and spatial-dominated features, respectively. The dynamic gating weights are expressed by the following formula:

(15) $$\left\{\begin{array}{c} \alpha =\sigma \left(GlobalAvgPool\left(F_{sp|s}\right)\cdot W_{g}\right)\\ \beta =\sigma \left(GlobalAvgPool\left(F_{s|sp}\right)\cdot W_{g}\right)\\ \alpha +\beta =1 \end{array}\right.$$

where σ is the sigmoid function, GlobalAvgPool is the global average pooling (compressing the spatial and spectral dimensions), and $W_{g}$ is a learnable parameter.

Weighted fusion:

(16) $$F_{fusion}=\alpha \cdot F_{sP|s}+\beta \cdot F_{s|sp}\in R^{k\times k\times B\times C^{\prime }}$$

Residual connection and dimension restoration: Add the fused features to the original expanded features (after dimensionality reduction) with residual connections, and restore to the input channel number *C*:

(17) $$F_{joint}=Conv(F_{fusion}+Conv(F_{s}^{Spatial}||F_{sp}^{spectral}))\in R^{k\times k\times B\times C}$$

where || represents channel concatenation, and *Conv* is a 1 × 1 × 1 convolution used for dimension adjustment.

### 3.3. Loss Function

To adapt to the core characteristics of DADFN, a multi-scale spectral–spatial collaborative constraint loss (MSSCC) function is designed. This loss function constructs collaborative constraints from three dimensions: the rationality of dynamic kernel generation, spectral consistency, and spatial detail fidelity. It ensures that the network decouples degradation information while considering the band correlation in the spectral dimension and multi-scale details in the spatial dimension of HSIs. Its mathematical definition is as follows:

(18) $$L_{MSSCC}=\alpha_{MSSCC}\cdot L_{DK}+\beta_{MSSCC}\cdot L_{MSpec}+\gamma_{MSSCC}\cdot L_{MSpat}$$

where $\alpha_{MSSCC,}\beta_{MSSCC}$, and $\gamma_{MSSCC}$ are weight coefficients satisfying $\alpha_{MSSCC}+\beta_{MSSCC}+\gamma_{MSSCC}=1$; $L_{DK}$ is the total dynamic kernel loss; $L_{MSpec}$ is the total multi-scale spectral loss; and $L_{MSpat}$ is the total multi-scale spatial loss.

#### 3.3.1. Total Dynamic Kernel Loss for Blur Kernel Dynamic Generation

The blur kernels of real images mostly follow a sparse distribution. In this paper, L1 regularization is used to constrain the sparsity of dynamic kernels, and its mathematical expression is as follows:

(19) $$L_{DK1}=\frac{1}{B\cdot k^{2}}\sum_{b=1}^{B}\sum_{i=1}^{k}\sum_{j=1}^{k}\left|K_{b,i,j}\right|$$

where B is the number of spectral bands, k is the size of the blur kernel, and $K_{b,i,j}$ is the (i, j)-th element of the blur kernel for the b-th band.

Since the degradation degree varies significantly across different bands of HSIs, to ensure that the generated kernels have band specificity and avoid all bands sharing similar kernels, $L_{DK2}$ is designed as a constraint, expressed by the following formula:

(20) $$L_{DK2}=\frac{1}{B\cdot (B-1)}\sum_{b=1}^{B}\sum_{b^{\prime }\neq b}^{B}\frac{{\left\|K_{b}-K_{b^{\prime }}\right\|}_{2}^{2}}{\max\left({\left\|K_{b}\right\|}_{2}^{2},{\left\|K_{b^{\prime }}\right\|}_{2}^{2}\right)+\varepsilon }$$

where $K_{b}$ is the flattened vector of the blur kernel for the b-th band; **ε** is a very small number used to prevent the denominator from being zero; ‖∙‖_2_ is the L2 norm.

Combining the above two formulas, the total dynamic kernel loss can be obtained:

(21) $$L_{DK}=L_{DK1}+w_{DK}\cdot L_{DK2}$$

The weight $w_{DK}$ is used to prevent either sparsity or difference from dominating excessively.

#### 3.3.2. Total Multi-Scale Spectral Loss

To ensure consistent spectral trends across different scales, the reconstructed image and the real image are each downsampled to generate three scales: the original scale *s*_0_, 1/2 scale *s*_1_, and 1/4 scale *s*_2_. The cosine similarity loss of the pixel-level spectral curves is calculated at each scale, as shown in the following formula:

(22) $$L_{MSpec1}=\frac{1}{S\cdot H_{s}\cdot W_{s}\cdot B}\sum_{s\in \{s_{0},s_{1},s_{2}\}}\sum_{h=1}^{H_{s}}\sum_{w=1}^{W_{s}}\left(1-\frac{R_{s,h,w}\cdot T_{s,h,w}}{{\left\|R_{s,h,w}\right\|}_{2}\cdot {\left\|T_{s,h,w}\right\|}_{2}+\varepsilon }\right)$$

where *S* = 3 is the number of scales, $H_{s}$ and $W_{s\;}are$ the spatial sizes of the s-th scale, $R_{s,h,w}$ is the spectral vector of the (h, w) pixel at the s-th scale of the reconstructed image, $T_{s,h,w}$ is the spectral vector at the corresponding position of the real image, and ε is a very small number used to prevent the denominator from being zero.

There is a strong correlation between the adjacent bands of HSIs. To avoid jumps between bands after reconstruction, an inter-band correlation constraint is established, as shown in the following formula:

(23) $$L_{MSpec2}=\frac{1}{B-1\cdot H\cdot W}\sum_{b=1}^{B-1}\sum_{h=1}^{H}\sum_{w=1}^{W}{\left((R_{h,w,b+1}-R_{h,w,b})-((T_{h,w,b+1}-T_{h,w,b})\right)}^{2}$$

where $R_{h,w,b}$ is the gray value of the (h, w)-th pixel in the b-th band of the reconstructed image, and $T_{h,w,b}$ is the corresponding value of the real image. Combining the above two formulas, the total multi-scale spectral loss can be obtained:

(24) $$L_{MSpec}=L_{MSpec1}+w_{MSpec}\cdot L_{MSpec2}$$

The weight $w_{MSpec}$ is used to prevent the correlation constraint from excessively suppressing the single-band accuracy.

#### 3.3.3. Total Multi-Scale Spatial Loss

Using the same *s*_0_, *s*_1_, and *s*_2_ scales as the $L_{MSpec}$, the SSIM loss at each scale is calculated to ensure that the spatial structure is preserved at different resolutions:

(25) $$L_{MSpat1}=\frac{1}{S\cdot B}\sum_{s\in \{s_{0},s_{1},s_{2}\}}\sum_{b=1}^{B}\left(1-SSIM(R_{s,b},T_{s,b})\right)$$

where $R_{s,b}$ is the single-band image of the b-th band at the s-th scale of the reconstructed image, and $T_{s,b}$ is the corresponding band image of the real image. SSIM (∙) is the structural similarity index, with a value range of 0–1, where 1 indicates complete consistency.

The edge maps of the reconstructed image and the real image are extracted using a 3 × 3 Sobel operator, and the L1 loss of the edge regions is calculated to focus on enhancing the restoration of edge details:

(26) $$L_{MSpat2}=\frac{1}{B\cdot H\cdot W}\sum_{b=1}^{B}\sum_{h=1}^{H}\sum_{w=1}^{W}\left|\nabla R_{b,h,w}-\nabla T_{b,h,w}\right|$$

where $\nabla R_{b,h,w}$ is the Sobel edge response value of the (*h, w*)-th pixel in the b-th band of the reconstructed image, and $\nabla T_{b,h,w}$ is the corresponding value of the real image.

From the above two formulas, the expression for the total multi-scale spatial loss can be derived:

(27) $$L_{MSpat}=L_{MSpat1}+w_{MSpat}\cdot L_{MSpat2}$$

The weight $w_{MSpat}$ is used to emphasize the importance of edge details.

## 4. Experiments

### 4.1. Datasets and Experimental Settings

#### 4.1.1. Dataset Construction

In this experiment, the public datasets CAVE [43] and Harvard [6] in the field of HSIs were used as benchmark datasets to verify the effectiveness of the proposed DADFN. Detailed information about the CAVE and Harvard datasets is shown in the following table (Table 1).

In the experiment, the images in the CAVE dataset and Harvard dataset are first subjected to degradation operations. Then, the 31 images in the CAVE dataset are divided into a training set, a validation set, and a test set in a ratio of 7:1:2. Specifically, the first 21 images are used as the training set, the subsequent four images are used as the validation set, and the last six images are used as the test set. The Harvard dataset contains 50 images, which are also divided into a training set, a validation set, and a test set in a ratio of 7:1:2. That is, the first 35 images are used as the training set, the subsequent five images are used as the validation set, and the last 10 images are used as the test set.

To simulate the real degradation process of HSIs, all HR images in the CAVE and Harvard datasets are degraded based on the dynamic degradation model described in Section 3.1 to generate corresponding LR images, thereby constructing LR–HR paired data for model training. Due to the large size of the original HR images, direct degradation easily leads to distortion of image edges and the inaccurate calculation of blur kernels. Therefore, before degradation, the images need to be cropped into non-overlapping sub-blocks of a fixed size. For the CAVE dataset, the 512 × 512 HR images are cropped into 16 non-overlapping sub-blocks with a step size of 128 and a size of 256 × 256, where each sub-block has a size of 256×256×31. For the Harvard dataset, the 1024 × 1024 HR images are cropped into 16 non-overlapping sub-blocks with a step size of 256 and a size of 512 × 512, where each sub-block has a size of 512×512×31. The degradation parameters of the HR images are set as follows: mixed Gaussian kernel with σ ∈ [0.5, 2.5], motion blur kernel with an angle range of [0°, 180°], and a length range of [5, 25]. The blur kernel parameters for each band are randomly sampled, and the kernel parameters at different spatial positions within the same band are smoothly changed through Gaussian interpolation. The downsampling hyperparameter s = 2 is used to simulate the resolution limitation of imaging equipment. Spectrally dependent base noise with σ ∈ [0.01, 0.05] and a signal-dependent coefficient in the range of [0.1, 0.3] are added.

After the above degradation operations, each degraded LR sub-block corresponds to an original HR image, forming the LR–HR pairs required for training.

#### 4.1.2. Experimental Settings

The code in this paper is based on the PyTorch 2.2.2 framework and uses the Adam optimizer. The optimizer adopts standard parameters. The first 200 epochs are set as the fast iteration stage with an initial learning rate of 1 × 10^−4^. From the 200th to the 300th epoch, a cosine annealing scheduling strategy is adopted to make the learning rate decrease smoothly and not lower than 1 × 10^−6^, entering the fine-tuning stage. During training, the batch size is set to 16 and the number of epochs is 300. To expand the number of training samples, data augmentation strategies including random horizontal flipping, random vertical flipping, and random 90-degree rotation are applied to the training samples in the CAVE dataset and Harvard dataset. As a result, the actual number of training samples in the CAVE dataset is 21 × 3 = 63 images, and that in the Harvard dataset is 35 × 3 = 105 images.

The computer used is equipped with a 28-core AMD EPYC 7453 CPU and two RTX 4090 GPUs with a total of 48.0 GB of video memory.

The hyper-parameter settings are as follows: For the MSSCC loss weights, to ensure the highest priority of spectral information in HSIs, the coefficient $\beta_{MSSCC}$ of the multi-scale spectral total loss $L_{MSpec}$ is set to 0.4. The coefficient $\alpha_{MSSCC}$ of the dynamic kernel total loss $L_{DK}$ and the coefficient $\gamma_{MSSCC}$ of the multi-scale spatial total loss $L_{MSpat}$ are equal, both set to 0.3, to ensure the coordinated optimization of spectral and spatial aspects. The weight of the dynamic kernel total loss $w_{DK}$ is set to 0.2; in the multi-scale spectral total loss function, the weight ${\;w}_{MSpec}$ is assigned a value of 0.5; and in the multi-scale spatial total loss function, the weight $w_{MSpec}$ is also set to 0.3. This weight configuration matches the multi-scale spectral total loss weight, balancing the spatial–spectral performance of the model.

#### 4.1.3. Evaluation Metrics

In this paper, the evaluation metrics are designed from three dimensions: spectral fidelity, spatial detail restoration, and overall quality. The experimental evaluation metrics include spectral angle mapper (SAM) [43], peak signal-to-noise ratio (PSNR) [43], structural similarity index (SSIM) [43], and spectral information divergence (SID) [43]. Let X be the true spectral vector and $\hat{X}$ be the high-resolution spectral vector. The calculation methods of the above evaluation metrics are as follows:

SAM: This is used to measure the directional difference between the super-resolved result and the true spectral vector. A smaller value indicates higher spectral fidelity. Its mathematical expression is [44]:

(28) $$SAM\left(X,\hat{X}\right)=arccos(\frac{x_{i,j}\cdot {\hat{x}}_{i,j}}{{\left||x_{i,j}|\right|}_{2}\cdot {\left|\left|{\hat{x}}_{i,j}\right|\right|}_{2}+\varepsilon })$$

where **ε** is a very small number used to prevent the denominator from being zero.

PSNR: This is a core quantitative metric for measuring the spatial pixel difference between the super-resolved result and the high-resolution true value. A higher value indicates better spatial fidelity of the super-resolved image and smaller pixel-level errors. Its mathematical expression is [44]:

(29) $$PSNR=10\cdot {log}_{10}(\frac{{MAX\left(X\right)}^{2}}{MSE(X,\hat{X})})$$

where $MAX\left(X\right)$ is the maximum value of the high-resolution true image X, and $MSE(X,\hat{X)}$ is the mean square error between $\hat{X}$ and X.

SSIM: This is a core metric for measuring the consistency of spatial structures between supper-resolution images and high-resolution images. A value closer to 1 indicates a higher degree of matching between the super-resolved image and the true value in terms of spatial structures such as textures and edges. Its mathematical expression is [45]:

(30) $$SSIM\left(X,\hat{X}\right)=\frac{\left(2\mu_{X}\mu_{\hat{X}}+C)(2\sigma_{X\hat{X}}+C_{2}\right)}{\left(\mu_{X}^{2}+\mu_{\hat{X}}^{2}+C_{1})(\sigma_{X}^{2}+\sigma_{\hat{X}}^{2}+C_{2}\right)}$$

where $\mu_{X}$ is the mean value of X in the local window, $\mu_{\hat{X}}$ is the mean value of $\hat{X}$ in the local window, $\sigma_{X}$ is the standard deviation of X in the local window, $\sigma_{\hat{X}}$ is the standard deviation of $\hat{X}$ in the local window, and $\sigma_{X\hat{X}}$ is the covariance between X and $\hat{X}$.

SID: This is a core quantitative metric for measuring the similarity of spectral distributions between supper-resolution images and high-resolution images. A smaller value indicates that the spectral distribution of the super-resolved image is closer to that of the true value. Its mathematical expression is [46]:

(31) $$SID\left(X,\hat{X}\right)=KL(P|\left|\hat{P}\right)+KL(P|\left|\hat{P}\right)$$

where ***P*** is the normalized vector of X, and $\hat{P}$ is the normalized vector of $\hat{X}$.

### 4.2. Experimental Results

In this experiment, the proposed DADFN algorithm is compared with four baseline algorithms from both quantitative evaluation and qualitative analysis perspectives. The quantitative evaluation uses four evaluation metrics: SAM, PSNR, SSIM, and SID. Among them, higher PSNR and SSIM values indicate better quality of the super-resolved image; smaller SAM and SID values indicate that the spectral distribution of the super-resolved image is closer to the true value. Three images are selected from both the CAVE dataset and the Harvard dataset, and the super-resolved images, locally enlarged super-resolved images, and original HR images are compared to determine the advantages and disadvantages of various comparative algorithms.

#### 4.2.1. Quantitative Evaluation Results

On the CAVE dataset and Harvard dataset, four quantitative evaluation metrics (SAM, PSNR, SSIM, and SID) are used to compare the DADFN algorithm proposed in this paper with the four baseline algorithms (EDSR [24], RCAN [22], DBSR [27], and KernelGAN [39]). The results are shown in Table 2. The optimal values of each evaluation metric are displayed in bold font, and the followings keep consistent. It can be seen from the data in the table that the DADFN algorithm proposed in this paper significantly outperforms the comparative algorithms in all metrics. Compared with KernelGAN, which achieves the best results among the comparative algorithms, the PSNR of the proposed DADFN algorithm is 34.52 dB, representing an improvement of 5%. Meanwhile, the SAM of DADFN is only 4.23, which is 32% lower than that of KernelGAN, proving that the DADFN algorithm proposed in this paper has better reconstruction capabilities for spatial and spectral information. The SSIM of DADFN is 0.958, which is 3% higher than that of KernelGAN, demonstrating that after super-resolution of HSIs, the algorithm in this paper is more consistent with the real situation in terms of spatial structure restoration. The SID of DADFN is 2.56, which is 47% lower than that of KernelGAN, proving that the algorithm proposed in this paper is closer to the actual value in terms of spectral restoration accuracy.

The Harvard dataset contains more natural and complex scenes. In this paper, the adaptability of the algorithm to spatially non-uniform blur and spectrally dependent noise is verified on the Harvard dataset. In the quantitative analysis experiment on the Harvard dataset, the four evaluation metrics (PSNR, SSIM, SAM, and SID) are still used for comparison, and the results are shown in Table 3. It can be seen from the table that the DADFN algorithm proposed in this paper still achieves the best performance in the quantitative comparison of the four metrics. Compared with KernelGAN, which performs the best among the comparative algorithms, the PSNR of the DADFN algorithm is 33.21 dB, an increase of 5% compared with KernelGAN. The SSIM of the DADFN algorithm is 0.943, which is 4% higher than that of KernelGAN. The SAM of the DADFN algorithm is 5.12, which is 26% lower than that of KernelGAN. The SID of the DADFN algorithm is 2.85, which is 47% lower than that of KernelGAN. The results in the table further prove that the HSIs obtained by the algorithm proposed in this paper in complex environmental degradation scenarios have super-resolved images with spatial and spectral information closer to the actual situation and better robustness.

#### 4.2.2. Qualitative Evaluation Results

**Figure 5:**
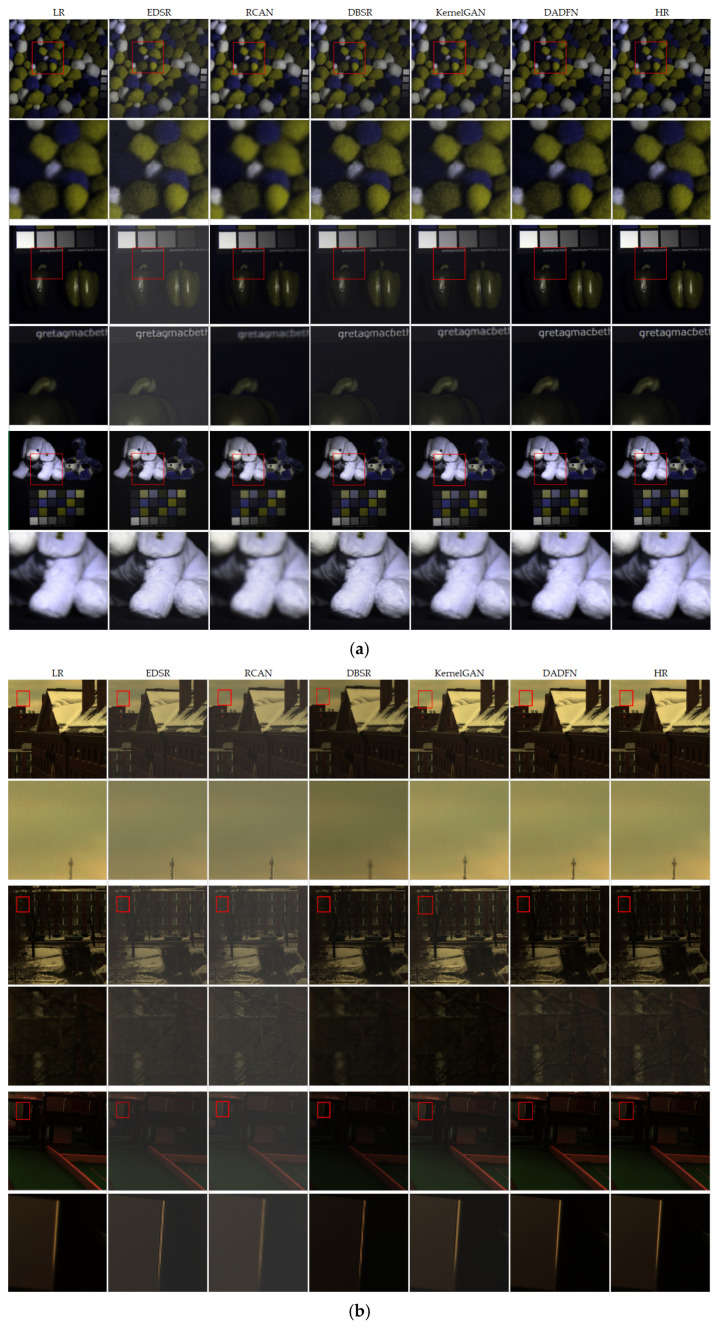
Qualitative comparative results. (**a**) Comparison between the proposed method and baseline methods on the CAVE dataset. (**b**) Comparison between the proposed method and baseline methods on the Harvard dataset.

To visually compare the experimental results, the algorithm proposed in this paper and the baseline algorithms are visualized. Figure 5 shows the image outputs of the proposed algorithm and the four baseline algorithms on the CAVE dataset and Harvard dataset, respectively. Group (a) presents the comparative results on the CAVE dataset. The EDSR algorithm has poor spectral fidelity, and the processed images are obviously overexposed. Although the RCAN, DBSR, and KernelGAN algorithms yield images with relatively good fidelity, the text becomes blurred and spatial details are distorted when the images are locally enlarged. Group (b) presents the comparative results on the Harvard dataset, which mainly consists of outdoor scenes; whether the outdoor images are bright or dark, the EDSR and RCAN algorithms have poor spectral fidelity, and the overall images are overexposed. In contrast, the DBSR and KernelGAN algorithms produce images that are overall underexposed. In terms of spatial details, the images processed by the four baseline algorithms show varying degrees of jagged edges and deformation of object contours when enlarged. The DADFN algorithm proposed in this paper achieves better spectral fidelity for both the CAVE dataset and the Harvard dataset and processes spatial details more delicately. The locally enlarged details are closer to the real images. Therefore, when the DADFN algorithm proposed in this paper is applied to the spatial super-resolution of HSIs, it can improve the spatial resolution while maximizing the retention of original spectral information and avoiding spectral distortion. It can meet the practical application requirement of HSIs, which is to obtain high-spatial and high-spectral images through super-resolution from hyperspectral data with low spatial resolution.

#### 4.2.3. Ablation Experiments

To verify the robustness and effectiveness of the DADFN algorithm proposed in this paper, ablation experiments are conducted on the DADFN and four baseline algorithms under different downsampling hyperparameters, as well as on different network modules of the DADFN.

##### Discussion on the Downsampling Hyperparameters

To verify the robustness of the DADFN algorithm and the four baseline algorithms under different degradation intensity scenarios and avoid one-sided conclusions caused by evaluation only under a single degradation intensity, ablation experiments are conducted in this paper under two degradation scenarios with downsampling hyperparameters of s = 2 and s = 4, and the PSNR and SID data are compared. The results are shown in Table 4. It can be seen from the data in the table that among the four baseline algorithms, KernelGAN performs the best. When the downsampling hyperparameter s changes from 2 to 4, the PSNR performance of KernelGAN decreases by 8% and the SID performance decreases by 46%. In contrast, the PSNR performance of the proposed DADFN algorithm only decreases by 1.8%, and the SID performance decreases by 25%. When the hyperparameters are s = 2 and s = 4, the experimental results of the DADFN algorithm proposed in this paper are compared with those of the baseline algorithms. The DADFN algorithm outperforms the four baseline algorithms in both PSNR and SID. The DADFN algorithm proposed in this paper exhibits better robustness and adaptability of core modules under different degradation intensity scenarios compared with the baseline algorithms. When s = 2 and s = 4, the experimental results of the DADFN algorithm proposed in this paper are compared with those of the baseline algorithms. The DADFN algorithm outperforms the four baseline algorithms in both PSNR and SID.

##### Discussion on the Effectiveness of Network Modules

To verify the effectiveness of the proposed degradation model based on the dynamic blur kernel generation network, ablation experiments are conducted by comparing the original DADFN model with models from which the DCS module, MLP module, and SSDCAF module are removed, respectively. The experimental results on the CAVE dataset are shown in Table 5. Compared with the models without the DCS module, MLP module, and SSDCAF module, the DADFN algorithm proposed in this paper shows significant improvements in various evaluation metrics, such as PSNR, SAM, and SID. This is because the high-dimensional degradation information is compressed into low-dimensional latent variables, and then the latent variables are decoded into dynamic kernels matching the input image through the generation network. This input-latent variable-dynamic kernel link enables the convolution kernel parameters of each region to be adaptively adjusted according to its real degradation state, fundamentally solving the adaptability defect of the one-size-fits-all static kernel.

##### Discussion on Loss Function Hyperparameters

During parameter tuning, adjustments were made based on experience within the ranges of α ∈ [0.2, 0.5], β ∈ [0.3, 0.5], and γ ∈ [0.2, 0.4]. The results are presented in Table 6. The results show that the hyperparameter values set in this paper are optimal, and within different ranges of hyperparameters, the PSNR fluctuation is ≤0.17 dB and the SAM fluctuation is ≤ 0.22 × 10^−2^ rad, proving that the model exhibits strong robustness to hyperparameters and can operate stably without requiring complex parameter tuning. The optimal value is indicated in bold.

For the dynamic kernel total loss hyperparameter $w_{DK}$, multi-scale spectral total loss function hyperparameter $w_{MSpec}$, and multi-scale spatial total loss function hyperparameter $w_{MSpat}$, experiments were conducted to verify the necessity of each hyperparameter by setting one parameter to 0 and the other two to the original data. The results are shown in Table 7.

The performance degradation is significant when $w_{MSpec}$ is missing (SAM increases by 2.66 × 10^−2^ rad), proving the core position of spectral fidelity. When $w_{DK}$ is missing, kernel constraints fail, and both spectral and spatial indicators decrease (PSNR decreases by 2.37 and SID increases by 0.02), proving the supporting role of dynamic verification in reconstruction. When $w_{MSpat}$ is missing, spatial details are lost (SSIM decreases by 0.043), proving the key role of spatial enhancement.

To verify robustness, we conducted small-scale fluctuations on the optimal $w_{DK}$, $w_{MSpec},\;and\;w_{MSpat}$, and the results are shown in the following table (Table 8).

From the data shown in the Table 8, it can be seen that for all combinations, PSNR fluctuates within the range of 34.20–34.52 dB, with a maximum decrease of only 0.32 dB and no significant performance degradation; SAM fluctuates within the range of 4.18–4.39 × 10^−2^ rad, with a maximum increase of 0.16 × 10^−2^ rad, and the spectral fidelity remains stable; SSIM fluctuates within the range of 0.952–0.958, with a maximum decrease of only 0.006, and there is no significant decrease in spatial structure similarity; and SID fluctuates within the range of 2.52–2.68 × 10^−2^, consistent with the trend of SAM changes. This indicates that the optimal weight has strong robustness within the specified range.

##### Complexity Analysis

To comprehensively evaluate the practicality of the proposed DADFN, this section conducts a quantitative complexity analysis of DADFN and baseline methods. The analysis includes three core metrics: parameter count, FLOPs, and training/inference time. All experiments are conducted under the same hardware and software environment.

The quantitative comparison results are shown in Table 9.

From the Table 9, we can see that DADFN has 78.9 M parameters, which is higher than EDSR (43.2 M) and DBSR (67.8 M) but lower than RCAN (82.6 M). Compared with KernelGAN (75.4 M), the increase is only 3.5 M (≈4.6%). The additional parameters of DADFN mainly come from the spectral–spatial dynamic cross-attention fusion module (≈6.2 M) and the multi-layer perceptron modules (≈3.8 M), which realize independent feature mapping. However, the parameter overhead is controlled by low-dimensional latent variable + dynamic kernel sharing.

**Figure 6:**
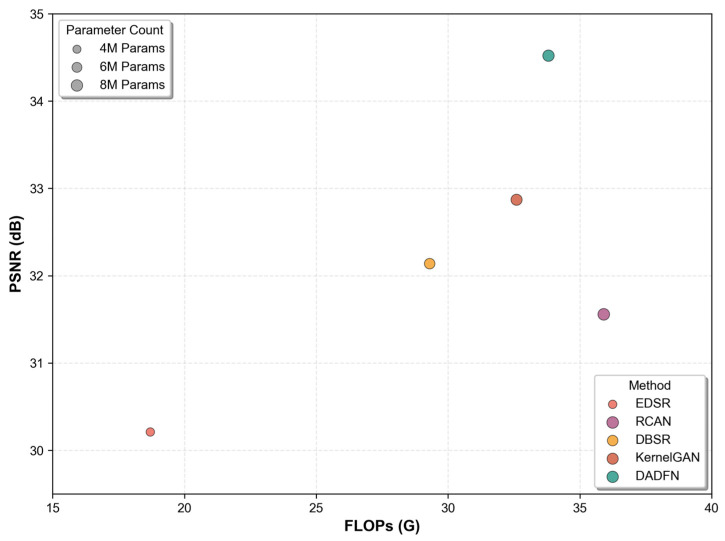
The scatter plot of the complexity of FLOPs.

DADFN’s FLOPs are 33.8 G, which is 80.7% higher than EDSR (18.7 G) but 5.9% lower than RCAN (35.9 G), and only 3.7% higher than KernelGAN (32.6 G). The main source of FLOPs in DADFN is the 3D dynamic convolution, but the computational cost is optimized by using 1 × 1 × 1 convolution for dimension adjustment and broadcast operations. As shown in Figure 6 with the scatter plot of the complexity of FLOPs, DADFN achieves the highest PSNR (34.52 dB), with FLOPs close to KernelGAN and RCAN, demonstrating a superior complexity–performance trade-off.

DADFN’s training time is 21.2 h, which is 72.3% longer than EDSR (12.3 h) but 2.3% shorter than RCAN (21.7 h) and 5.5% longer than KernelGAN (20.1 h). The slight increase compared to KernelGAN is due to the dynamic kernel generation process, but the gap is controlled within 1 h.

DADFN’s inference time is 44.6 ms per image, which is slower than EDSR (28.5 ms) and DBSR (39.7 ms) but faster than RCAN (45.2 ms) and comparable to KernelGAN (42.3 ms).

## 5. Conclusions

To address the core problem of high super-resolution difficulty of HSIs caused by dynamic degradation characteristics, we propose a DADFN for SSR. A DCSM is designed to achieve decoupled encoding of spectral and spatial degradation information in latent variables. Combined with an independent MLP module, feature dimension mapping is completed. Finally, a SSDCAF module is used to establish a dynamic connection between the two-dimensional degradation information, generating 3D dynamic blur kernels adapted to different bands and spatial positions. This design fundamentally breaks through the limitation of the one-size-fits-all static kernel in traditional methods, enabling degradation modeling to accurately match the physical characteristics of HSIs, such as the dynamic nature in the spectral dimension and the inhomogeneity in the spatial dimension.

The DADFN algorithm proposed in this paper achieved superior super-resolution results compared to the baseline algorithm for both indoor and outdoor public datasets. Especially on the Harvard dataset, the SID metric increased by 47% compared to the SOTA algorithm.

Since this paper only focuses on the performance of the DADFN algorithm in terms of spectral fidelity and spatial detail restoration after SSR, it does not consider the computational complexity or modeling ability for extreme degradation scenarios. In the future, the computational cost will be reduced through lightweight 3D convolution and model distillation. Attention-weighted latent variables will be introduced to enhance the modeling ability for extreme degradation. Multi-branch latent variables will be designed to expand the adaptation range of degradation types, further improving the practical value of the method.

## Figures and Tables

**Table 1:** Detailed information of the CAVE and Harvard datasets.

Dataset	Number of Images	Spatial Resolution	Spectral Range (nm)
CAVE	32	512 × 512	400–700
Harvard	50	1024 × 1024	420–720

**Table 2:** Comparison of verification results on the CAVE dataset.

Baseline Methods	PSNR (dB)	SSIM	SAM (×10^−2^ Rad)	SID (×10^−2^)
EDSR	30.21	0.892	8.76	6.21
RCAN	31.56	0.915	7.50	5.83
DBSR	32.14	0.923	6.89	5.12
KernelGAN	32.87	0.931	6.15	4.87
**DADFN**	**34.52**	**0.958**	**4.23**	**2.56**

**Table 3:** Comparison of verification results on the Harvard dataset.

Baseline Methods	PSNR (dB)	SSIM	SAM (×10^−2^ Rad)	SID (×10^−2^)
EDSR	28.93	0.867	9.54	7.12
RCAN	30.15	0.889	8.21	6.53
DBSR	30.87	0.896	7.58	5.98
KernelGAN	31.62	0.908	6.93	5.42
**DADFN**	**33.21**	**0.943**	**5.12**	**2.85**

**Table 4:** Ablation experiments under two degradation scenarios with downsampling hyperparameters of s = 2 and s = 4.

	s = 2	s = 2	s = 4	s = 4
	PSNR (dB)	SID (×10^−2^)	PSNR (dB)	SID (×10^−2^)
EDSR	30.21	6.21	27.58	9.87
RCAN	31.56	5.83	28.93	8.92
DBSR	32.14	5.12	28.45	7.65
KernelGAN	32.87	4.87	30.12	7.13
**DADFN**	**34.52**	**2.56**	**33.87**	**3.21**

**Table 5:** Ablation experiment on the effectiveness of dynamic kernel generation controlled by latent variables.

Method	PSNR (dB)	SSIM	SAM (×10^−2^ Rad)	SID (×10^−2^)
**DADFN**	**34.52**	**0.958**	**4.23**	**2.56**
No DCS	29.93	0.865	6.75	6.02
No MLP	31.12	0.901	4.80	3.25
No SSDCAF	30.21	0.878	5.52	4.11

**Table 6:** Hyperparameter sensitivity analysis.

Hyperparameter Combination	PSNR (dB)	SSIM	SAM (×10^−2^ Rad)	SID (×10^−2^)
α = 0.2, β = 0.3, γ = 0.4	34.36	0.951	4.33	2.63
α = 0.2, β = 0.4, γ = 0.4	34.38	0.953	4.31	2.60
α = 0.2, β = 0.5, γ = 0.3	34.43	0.955	4.35	2.60
α = 0.2, β = 0.6, γ = 0.2	31.12	**0.958**	4.30	2.58
α = 0.3, β = 0.3, γ = 0.4	34.48	0.951	4.28	**2.56**
**α = 0.3, β = 0.4, γ = 0.3**	**34.52**	**0.958**	**4.23**	**2.56**
α = 0.3, β = 0.5, γ = 0.2	34.51	0.954	4.24	2.67
α = 0.4, β = 0.3, γ = 0.3	34.48	0.956	4.32	2.71
α = 0.5, β = 0.3, γ = 0.2	34.41	0.956	4.41	2.69

**Table 7:** Single sensitivity analysis of three hyperparameters $w_{DK}$, $w_{MSpec}$, and $w_{MSpat}$.

Hyperparameter Combination	PSNR (dB)	SSIM	SAM (×10^−2^ Rad)	SID (×10^−2^)
$w_{DK}=0,\;w_{MSpec}=0.5,\;w_{MSpat}=0.3\;$	33.78	0.958	4.76	2.61
$w_{DK}=0.2,\;w_{MSpec}=0,\;w_{MSpat}=0.3$	32.15	0.963	6.89	2.58
$w_{DK}=0.2,\;w_{MSpec}=0.5,\;w_{MSpat}=0$	33.92	0.915	4.18	2.60
$w_{DK}=0.2,w_{MSpec}=0.5,w_{MSpat}=0.3$	**34.52**	**0.958**	**4.23**	**2.56**

**Table 8:** Weight robustness analysis.

Hyperparameter Combination	PSNR (dB)	SSIM	SAM (×10^−2^ Rad)	SID (×10^−2^)
$w_{DK}=0.1,\;w_{MSpec}=0.5,\;w_{MSpat}=0.4$	34.48	0.957	4.26	2.56
$w_{DK}=0.1,\;w_{MSpec}=0.6,\;w_{MSpat}=0.3$	34.42	0.955	4.18	2.56
$w_{DK}=0.2,\;w_{MSpec}=0.4,\;w_{MSpat}=0.4$	34.35	0.955	4.32	2.60
$w_{DK}=0.2,\;w_{MSpec}=0.5,\;w_{MSpat}=0.3$	**34.52**	**0.958**	**4.23**	**2.56**
$w_{DK}=0.2,\;w_{MSpec}=0.6,\;w_{MSpat}=0.2$	34.40	0.954	4.23	2.60
$w_{DK}=0.3,\;w_{MSpec}=0.6,\;w_{MSpat}=0.1$	34.20	0.952	4.39	2.68
$w_{DK}=0.3,\;w_{MSpec}=0.5,\;w_{MSpat}=0.2$	34.25	0.953	4.36	2.65

**Table 9:** Complexity comparison.

Method	Parameter Count (M)	FLOPs (G)	Training Time (h)	Inference Time (ms)
EDSR	43.2	18.7	12.3	28.5
RCAN	82.6	35.9	21.7	45.2
DBSR	67.8	29.3	18.5	39.7
KernelGAN	75.4	32.6	20.1	42.3
DADFN	78.9	33.8	21.2	44.6

## Data Availability

The raw data supporting the conclusions of this article will be made available by the authors on request.

## References

[1] Alahmari S., Yonbawi S., Racharla S., Lydia E.L., Ishak M.K., Alkahtani H.K., Aljarbouh A., Mostafa S.M. (2025). Hybrid Multi-Strategy Aquila Optimization with Deep Learning Driven Crop Type Classification on Hyperspectral Images. Comput. Syst. Sci. Eng..

[2] Efrat N., Glasner D., Apartsin A., Nadler B., Levin A. Accurate blur models vs. image priors in single image super-resolution. Proceedings of the IEEE International Conference on Computer Vision.

[3] Cao J., Cao Y., Pang L., Meng D., Cao X. (2024). Hair: Hypernetworks-based all-in-one image restoration. arXiv.

[4] Khonina S.N., Kazanskiy N.L., Oseledets I.V., Nikonorov A.V., Butt M.A. (2024). Synergy between artificial intelligence and hyperspectral imagining—A review. Technologies.

[5] Maiseli B., Abdalla A.T. (2024). Seven decades of image super-resolution: Achievements, challenges, and opportunities. EURASIP J. Adv. Signal Process..

[6] Wang Q., Li Q., Li X. (2020). Hyperspectral image superresolution using spectrum and feature context. IEEE Trans. Ind. Electron..

[7] Liu T., Liu Y., Zhang C., Yuan L., Sui X., Chen Q. (2024). Hyperspectral image super-resolution via dual-domain network based on hybrid convolution. IEEE Trans. Geosci. Remote Sens..

[8] Ranjan P., Girdhar A. (2022). A comprehensive systematic review of deep learning methods for hyperspectral images classification. Int. J. Remote Sens..

[9] Wang H., Quan S., Liu J., Xiao H., Peng Y., Wang Z., Li H. (2025). Progressive multi-scale multi-attention fusion for hyperspectral image classification. Sci. Rep..

[10] Li J., Wang H., Li Y., Zhang H. (2025). A Comprehensive Review of Image Restoration Research Based on Diffusion Models. Mathematics.

[11] Zhang W., Shi G., Liu Y., Dong C., Wu X.M. A closer look at blind super-resolution: Degradation models, baselines, and performance upper bounds. Proceedings of the IEEE/CVF Conference on Computer Vision and Pattern Recognition.

[12] Wang J., Xiang L., Liu L., Xu J., Li P., Xu Q., He Z. (2024). Towards Real-World Remote Sensing Image Super-Resolution: A New Benchmark and an Efficient Model. IEEE Trans. Geosci. Remote Sens..

[13] Zhang K., Liang J., Van Gool L., Timofte R. Designing a practical degradation model for deep blind image super-resolution. Proceedings of the IEEE/CVF International Conference on Computer Vision.

[14] Kumar A., Kashyap Y., Sharma K.M., Vittal K.P., Shubhanga K.N. (2025). MSSEAG-UNet: A Novel Deep Learning Architecture for Cloud Segmentation in Fisheye Sky Images and Solar Energy Forecast. IEEE Trans. Geosci. Remote Sens..

[15] Wang Z., Cao X., Yao Y., Feng L., Qin H. (2025). Segmentation of Green Roofs in High-Resolution Remote Sensing Images with GR-Net. IEEE Trans. Geosci. Remote Sens..

[16] Patnaik A., Bhuyan M.K., Alfarhood S., Safran M. (2025). Hyperspectral Image Super-Resolution via Grouped Second-Order Spatial Features and Spectral Attention Network. IEEE J. Sel. Top. Appl. Earth Obs. Remote Sens..

[17] Liu S., Zhangn J., Zhang Z., Hu S., Xiao B. (2025). Ground-Based Remote Sensing Cloud Image Segmentation Using Convolution-MLP Network. IEEE J. Sel. Top. Appl. Earth Obs. Remote Sens..

[18] Zhang J., Qu H., Jia J., Li Y., Jiang B., Chen X., Peng J. (2025). Multi-scale Spatial-Spectral CNN-Transformer Network for Hyperspectral Image Super-Resolution. IEEE J. Sel. Top. Appl. Earth Obs. Remote Sens..

[19] Zhao G., Wu H., Luo D., Ou X., Zhang Y. (2024). Spatial spectral interaction super-resolution cnn-mamba network for fusion of satellite hyperspectral and multispectral image. IEEE J. Sel. Top. Appl. Earth Obs. Remote Sens..

[20] Zhang L., Nie J., Wei W., Li Y., Zhang Y. (2020). Deep blind hyperspectral image super-resolution. IEEE Trans. Neural Netw. Learn. Syst..

[21] Yue Z., Zhao Q., Xie J., Zhang L., Meng D., Wong K.Y.K. Blind image super-resolution with elaborate degradation modeling on noise and kernel. Proceedings of the IEEE/CVF Conference on Computer Vision and Pattern Recognition.

[22] Wu G., Jiang J., Jiang J., Liu X. (2024). Transforming image super-resolution: A convformer-based efficient approach. IEEE Trans. Image Process..

[23] Xu H., Quan Y., Qin M., Wang Y., Fang C., Li Y., Zheng J. (2025). Nonlinear Learnable Triple-Domain Transform Tensor Nuclear Norm for Hyperspectral Image Super-Resolution. IEEE Trans. Geosci. Remote Sens..

[24] Lim B., Son S., Kim H., Nah S., Mu Lee K. Enhanced deep residual networks for single image super-resolution. Proceedings of the IEEE Conference on Computer Vision and Pattern Recognition Workshops.

[25] Jenefa A., Kuriakose B.M., Edward Naveen V., Lincy A. (2023). EDSR: Empowering super-resolution algorithms with high-quality DIV2K images. Intell. Decis. Technol..

[26] Huang Y., Jiang Z., Lan R., Zhang S., Pi K. (2021). Infrared image super-resolution via transfer learning and PSRGAN. IEEE Signal Process. Lett..

[27] Yang P., Ma Y., Mei X., Chen Q., Wu M., Ma J. (2025). Deep blind super-resolution for hyperspectral images. Pattern Recognit..

[28] Liu D., Li J., Yuan Q. (2021). A spectral grouping and attention-driven residual dense network for hyperspectral image super-resolution. IEEE Trans. Geosci. Remote Sens..

[29] Naganuma K., Ono S. (2024). Toward robust hyperspectral unmixing: Mixed noise modeling and image-domain regularization. IEEE J. Sel. Top. Appl. Earth Obs. Remote Sens..

[30] Akewar M., Chandak M. (2024). Hyperspectral imaging algorithms and applications: A review. TechRxiv.

[31] Wu C., Li J., Song R., Li Y., Du Q. (2023). HPRN: Holistic prior-embedded relation network for spectral super-resolution. IEEE Trans. Neural Netw. Learn. Syst..

[32] Mai G., Lao N., Sun W., Ma Y., Song J., Meng C., Ermon S. (2023). Learning continuous image representation for spatial-spectral super-resolution. arXiv.

[33] Parmar M., Lansel S., Wandell B.A. Spatio-spectral reconstruction of the multispectral datacube using sparse recovery. Proceedings of the IEEE International Conference on Image Processing.

[34] Palsson F., Sveinsson J.R., Ulfarsson M.O. (2017). Multispectral and hyperspectral image fusion using a 3-D-convolutional neural network. IEEE Geosci. Remote Sens. Lett..

[35] Lee C.M., Cheng C.H., Lin Y.F., Cheng Y.C., Liao W.T., Yang F.E., Wang Y.C.F., Hsu C.C. (2024). Prompthsi: Universal hyperspectral image restoration framework for composite degradation. arXiv.

[36] Li J., Cui R., Li B., Song R., Li Y., Dai Y., Du Q. (2020). Hyperspectral image super-resolution by band attention through adversarial learning. IEEE Trans. Geosci. Remote Sens..

[37] Zhang Y., Liang S., Li W., Ma H., Xu J., Ma Y., Xia X.G. (2025). UniTS: Unified Time Series Generative Model for Remote Sensing. arXiv.

[38] Cai Y., Lin J., Wang H., Yuan X., Ding H., Zhang Y., Gool L.V. (2022). Degradation-aware unfolding half-shuffle transformer for spectral compressive imaging. Adv. Neural Inf. Process. Syst..

[39] Park J., Kim H., Kang M.G. (2023). Kernel estimation using total variation guided GAN for image super-resolution. Sensors.

[40] Qin L., Huang X., Dong Q.L., Tang Y. (2025). Accelerated Douglas-Rachford splitting algorithm using neural net-work. Commun. Nonlinear Sci. Numer. Simul..

[41] Gu J., Lu H., Zuo W., Dong C. Blind super-resolution with iterative kernel correction. Proceedings of the IEEE/CVF Conference on Computer Vision and Pattern Recognition.

[42] Huang Y., Li S., Wang L., Tan T. (2020). Unfolding the alternating optimization for blind super resolution. Adv. Neural Inf. Process. Syst..

[43] Cao X., Lian Y., Liu Z., Zhou H., Wang B., Hunag B., Zhang W. (2023). Universal high spatial resolution hyperspectral imaging using hybrid-resolution image fusion. Opt. Eng..

[44] Khan M.M. (2019). High Dynamic Range Image Deghosting Using Spectral Angle Mapper. Computers.

[45] Michel J., Kalinicheva E., Inglada J. (2025). Revisiting remote sensing cross-sensor Single Image Super-Resolution: The overlooked impact of geometric and radiometric distortion. IEEE Trans. Geosci. Remote Sens..

[46] Gong J., Huang Z., Yang Z., Ding X., Li F. (2025). Spectral Information Divergence-Driven Diffusion Networks for Hyperspectral Target Detection. Appl. Sci..

